# Book Reviews

**Published:** 1813-09

**Authors:** 


*Medico-Chirurgical Transactions.* Published by the Medical and Chirurgical Society of London. Vol. III. 8vo. Lond. 1812.

(Continued from p. 170.)

Art. XVI. *A general View of the Composition of Animal Fluids. By* J. Berzelius, M.D.

Of this long article, on the Composition and Chemical Properties of Animal Fluids, by the Professor of Chemistry in the College of Medicine at Stockholm, we gave such an account in our last Half-yearly Report, as will preclude the necessity of going into it here. It contains analytical researches on the blood, in all its constituent parts—the chemical properties of fibrin—the chemical properties of the coloring matter—an inquiry into the influence of the iron, as producing its color, contained in the coloring matter—the serum—the albumen—and salts of the blood. It examines, by chemical analysis, the secreted fluids, viz. bile, saliva, the mucus of mucous membranes, the fluids of serous membranes, the humors of the eye; also the excreted fluids, viz. the fluid of perspiration, urine, and milk.

As a specimen of the results of the author's inquiry, his analysis of urine is inserted.

1000 parts of urine are composed of

| | |
|---|---|
| Water | 933·00 |
| Urea | 30·10 |
| Sulphate of potass | 3·71 |
| ——— of soda | 3·16 |
| Phosphate of soda | 2·94 |
| Muriate of soda | 4·45 |
| Phosphate of ammonia | 1·65 |
| Free lactic acid<br>Lactate of ammonia<br>Animal matter soluble in alcohol, and usually accompanying the lactates<br>Animal matter insoluble in alcohol<br>Urea not separable from the preceding | 17·14 |
| Earthy phosphates with a trace of fluate of lime | 1·00 |
| Uric acid | 1·00 |
| Mucus of the bladder | 0·32 |
| Silex | 0·03 |
| | 1000·00 |

ART. XVII. *A Case of Fungus Hæmatodes. By* GEORGE LANGSTAFF, Surgeon.

A perspicuous history of the progress of this dreadful disease, as it extended nearly to the whole frame. It began on the left shoulder, just below the spine of the scapula, at least it was visible first there, in the form of a tumor, about the size of a cherry, of a bluish red color. From thence it extended to the left axilla, where was the main bulk of the disease. Dissection discovered it on the sternum, in the liver, the pancreas, in the colon and the cœcum, the lungs, the pleura-pulmonalis, on the cranium beneath the pericranium, and on the dura mater, beneath the occipital bone.

What the peculiarity of the idiosyncracy is that generates this disease, or what methods can be employed to stop or retard its fatal progress, remain yet in obscurity. The subject of this case was a boot-maker, about 30 years of age, of middle stature, rather corpulent, *and of that sallow complexion peculiar to a female whose constitution has been impaired by long obstruction of the catamenia.*

ART. XVIII. *History of a severe Affection of the Organs of Respiration, with the Appearances on Dissection, and Remarks. By* A. P. WILSON PHILIP, M.D.

The causes producing this singular and fatal case of dyspnœa, are left, both by observation of the symptoms, and examination examination *post mortem*, in great obscurity; as well as is the *modus operandi* of emetics in producing the extraordinary relief of symptoms. How far the deficient carbonization of the blood was a cause or effect is doubtful; or if an effect, how far it might also become a cause aggravating symptoms and hurrying on the final termination, we fear must be left in a state of similar indecision. As a record of facts, and totally independent of opinions, this communication may be looked to as a valuable document.

ART. XIX. *An Account of a new Mode of Treatment in Chronic Rheumatism, and especially in Sciatica. By* ALEXANDER MARCET, M.D. F.R.S.

The value of this article, if the case is fairly related, and we feel no hesitation in believing it so to be, will place it very high in the scale of the *Methodus Medendi.* It consists in a peculiar mode of exciting perspiration, and is prefaced by some sensible remarks.

"I have frequently had the opportunity of observing, for the last six or seven years," says Dr. Marcet, "that the profuse and unavailing sweats which often spontaneously take place in the early stages of rheumatism, and exhaust the strength of the patients without alleviating their sufferings, are almost in every instance checked, and the pains proportionally relieved, by the use of antimonial medicines. The explanation which I ventured to offer of this paradoxical result, was, that the profuse flow of moisture from the pores, is not, in itself, the circumstance which diminishes pain in rheumatic affections; but that the relief is produced by a certain condition of the surface, or peculiar action of the cutaneous vessels, though generally productive of moisture, is not necessarily connected with profuse perspiration. It is this peculiar action which antimonials are so apt to promote; and there is no difficulty in conceiving, how the violent and colliquative paroxysms which occur in rheumatism, gradually yield to this gentle and uniform operation."

The new mode of treating chronic rheumatism, is *exciting perspiration by muscular action, with an increased quantity of clothing.* It was suggested to the patient, who relates his own case, by a celebrated race-horse (Vandyk) having been cured of a disorder which had all the symptoms of rheumatism, *by sweating in body-cloths.* The writer of this history had suffered several years by rheumatism, and particularly by that form of it denominated sciatica, when the above fact came to his knowledge. Having determined to pursue the method, which is similar to that employed by the Newmarket riders for the reduction of their weight,

"I clothed myself," the writer says, "in a sufficient quantity of flannel, and set out to walk as far and as fast as I could. With the utmost difficulty I proceeded half a mile, and the pain I suffered contributed not a little to the effect of the exercise in promoting perspiration. I returned home in a profuse sweat, rubbed myself dry before a fire, and went to bed. In about an hour I got up, found myself very much fatigued, but in other respects not worse. Forty-eight hours after this, I repeated the same kind of exercise, and found that I could walk a mile with as much ease as I had walked half that distance on the first day. My general sensations were the same as before; but, as the fatigue diminished, I thought I could perceive an amendment in my rheumatic pains. Two days afterwards I took a third walk, proceeded as before, and after it had a better night, less interrupted by pain than any I had enjoyed for eighteen months. Every succeeding walk has diminished my sufferings, and I may safely say that, after the sixth, I was as free from pain as I had ever been in my life. The only remnant I have left to remind me I was so lately a cripple, is a weakness in the left leg, particularly about the ankle, together with now and then a slight sensation of numbness along the sciatic nerve. I usually proceed to my sweating walks in the following manner: next to my skin I wear stockings, drawers, and a shirt, all of fleecy hosiery; over these I put one, two, or three, flannel drawers; one, two, or three, flannel waistcoats; and round my hips and loins I gird six yards of thick flannel; making, beside the drawers and waistcoats, eight thicknesses of flannel on the chief seat of the pain, and the origin of the sciatic nerve: over all this I wear warm pantaloons, and a great coat. When I have walked one or two miles, more or less, according to the heat of the day, I am generally in a profuse perspiration. I do not perceive that the quantity I perspire, has any influence on the efficacy of the remedy. I imagine that a violent action produced in the general system is the chief cause of its salutary effect. In consequence of this opinion, I cease the exercise the moment that a very increased action is well established. This is fully produced with the above quantity of clothing in moderately warm weather, by walking from one to two miles. When the excitement is well established, I find my pulse rise to between 90 and 100, and it is full and strong."

For a minute detail of particular circumstances, and a table of the variations of weight during this process, we must refer to the volume itself.

Art. XX. *Appendix to the Paper on Cynanche Laryngea. By* J. R. Farre, M.D.

This is a valuable addition to Dr. Farre's former paper; a distinction between *Cynanche Laryngea*, and *Cynanche Trachealis*, is its principal object.

"In *Cynanche laryngea* the symptoms are, uneasy sensations in the larynx, difficult and painful deglutition, partial swelling in the fauces, a supervening and perpetually increasing difficulty of breathing, inflammatory fever. In the *Cynanche trachealis* there is a difficulty of breathing, without any swelling in the fauces, or painful deglutition; glutition; the expirations, especially in coughing, are very shrill, the fever is inflammatory. In both the voice is changed, and in extreme cases is suppressed; the termination is by suffocation.

"The following are the morbid appearances. In *Cynanche laryngea,* the mucous membrane investing the epiglottis and the margin of the glottis is inflamed, serum is effused under it, or coagulable lymph on its external surface, by which the rima glottidis is narrowed, or actually closed. In *Cynanche trachealis,* the mucous membrane of the larynx and trachea is inflamed, and a layer of coagulated lymph is formed on its internal surface, from the extremity of the epiglottis to an indefinite extent within the trachea, by which the tube itself is narrowed or actually closed. A puriform fluid, instead of mucus, is found in the trachea and bronchia."

ART. XXI. *Some Remarks on the Use of Nitrate of Silver, for the detecting of minute Portions of Arsenic. By* ALEX. MARCET, M.D. F.R.S.

The general subject of this paper has had a full discussion in our Journal, particularly as respected the claim Mr. Hume of Long-acre had to the discovery of this test. The nature of the dense yellow precipitate which is produced by the application of minute quantities of solutions of ammonia and nitrate of silver, where the smallest quantity of arsenic is present, is the object of these Remarks; and is an answer to some objections made against this test by Mr. Sylvester of Derby, and published in Nicholson's Journal, vol. xxxiii. p. 306.

ART. XXII. *History of a Case of Remitting Ophthalmia, and its successful Treatment by Opium. By* JAMES CURRY, M.D. F.A.S.

This is an elaborate detail of the author's own case. It is singular in the extraordinary degree of pain with which it was accompanied, and in the remarkable efficacy of opium taken in very large doses. As a specimen of bold and decisive practice, and ingenious reasoning, Dr. Curry's paper will not disappoint the reader.

---

*A Practical Treatise on the Remittent Fever of Infants, with Remarks on Hydrocephalus Internus, or Water in the Brain, and several other Diseases; and Cases and Observations designed to illustrate the Influence exerted by a certain disordered State of the Chylopöietic Viscera upon local and constitutional Diseases, and to prove the utility and necessity of removing it, in order to facilitate and establish their Cure. By* JAMES MILLMAN COLEY, Member of the Royal College of Surgeons in London, &c. 8vo. pp. 156. Underwood and Longman and Co. 1813.

THE disease treated of in this publication is familiar to practitioners, practitioners, though we doubt whether all who have noticed it, regarded or treated it as fever. We do not, in fact, consider the name a very appropriate one, but it may serve for the nursery; and the author has much stronger pretensions to reputation than those which he might derive from naming a disease which has been termed marasmus, consumption, worm fever, bilious fever, typhus, &c. according to the taste or the judgment of the writer. Now, in our opinion, these are names of diseases entirely distinct from each other, and we also conceive we frequently meet with a remittent fever very different from the complaint before us. In some respects then, it is advantageous for a man not to be burthened with much reading: he may more securely investigate the case presented to him as it really appears, without being disturbed by names and authorities that might shake his judgment. What unlearned man, for instance, seeing a child whose appetite and strength had *gradually* sunk, with an inaptitude for exertion, irregularity in the bowels, and a wasting of the whole body, would think of *typhus* fever, or indeed of any sort of fever? But such is the commencement of the *remittent fever*.

"After these symptoms have continued for some time, the patient has several accessions of slight fever, more particularly towards evening; during which he evinces a strong propensity to sleep, seeks a recumbent posture, and is exceedingly peevish. The tongue at this period has seldom an unhealthy appearance, because digestion is not yet completely suspended. The pulse is an hundred or more in a minute.

"In this situation the patient will sometimes continue during several weeks, and at others will be suddenly attacked towards evening with a more violent paroxysm of fever; which is frequently considered by the parents to be the commencement of his disease. It is generally preceded by a shivering fit and vomiting, but seldom terminates with perspiration, the skin being remarkably dry through nearly the whole course of the complaint. The pulse during the paroxysm beats from an hundred and thirty to an hundred and sixty in a minute, and the respiration is performed with corresponding velocity. The cheeks are flushed, and the sleepiness is increased to an extreme degree, but is frequently interrupted with starts, expressions of pain about the belly, slight delirium, and sometimes with convulsions. A cough is noticed at this time, which generally continues through the whole of the illness, together with an almost constant picking of the skin about the eyes, nose, lips, and fingers.

"The duration of the febrile paroxysm is usually one or two hours, but in some instances will extend through the whole night, after which a remission takes place, and the patient becomes more wakeful and inclined for amusement, or it will sometimes terminate in sleep of a refreshing nature. The pulse now beats from an hundred and twenty to an hundred and thirty.

"The

"The return of these exacerbations is uncertain: most commonly there is one in the forenoon, one in the afternoon, and one in the night. The last is usually the longest and most violent. When the fever runs very high, we have much difficulty in observing any distinct remissions.

"There is much variation in the temperature of the body, the head, belly, and palms of the hands being more hot than any other parts on the surface.

"In some instances, the 'head is more affected even to a degree of raving, and one or other of the excretions is always remarkably increased. After this the patient becomes quieter than usual, says little, complains of nothing, and is not disposed to answer questions. He seldom asks for any thing, but in general takes his food or drink when it is offered him. The trunk of his body keeps to one posture, and he rarely moves his lower limbs; but his arms or hands are almost constantly in motion when he is awake. Sometimes he is flinging about his arms; sometimes he lies with his hands stretched down on the lower part of his belly, and his knees drawn up. At other times he is much employed in picking, not only his nose and lips, but even his tongue, eyes, and other parts of his face, till they become sore and chopped; and he gapes that he may reach his tongue, for he has not the power of putting it out of his mouth. At last his indifference as to answering questions ends in an impossibility of giving answers, for he is deprived both of speech and voice; and his jaws, in some cases, are so locked that nothing but liquids can be got into his mouth, and these with a good deal of difficulty. At this period, which seems to be the height of the disease, he slumbers, and is most composed, as usual, during the exacerbations; and in the remissions he performs the same gesticulations. From the time that there are settled symptoms of lowness, his eyes are reddish, dull, and inattentive; his countenance is marked with distress; his tongue, gums, teeth, and lips, are covered with a blackish fur; he is particularly uneasy before stools, or great explosions of wind; his urine and stools are involuntary, and yet he is quite sensible.'

"'The state of the belly is uncertain; but the stools are always unnatural, either as to their color, consistence, contents, or smell. Most commonly they are morbid in all these respects, for they are either whiter or darker than natural: they are always more offensive, are seldom without a great deal of slime, and sometimes consist of nothing but slime.'*

"'Digestion seems perfectly at a stand, for the food which is taken into the stomach will often be brought up unaltered, though it shall have remained down a considerable time. The intestines also seem to be in a manner paralysed: they exert no action on the food, for it passes off like a mass of putrid vegetable and animal matter, which has been some time subjected to heat and moisture, without its having

* "'Treatise on the Infantile Remittent Fever, by W. Butter, M.D.'—Callow, London."

the smallest resemblance, either in appearance or smell, to those fæces, where the powers of digestion have been exerted.

" 'When the disease has continued some time, the appetite is so totally destroyed, that for six or eight days together I have known the whole nourishment consist of about half a pint of toast and water in the twenty-four hours.'*

" I have frequently known that the patient has taken nothing but water, excepting his medicines, for four or five weeks together, and yet has ultimately recovered."

These are the leading and most usual symptoms of the complaint. But Mr. Coley has noticed some others, as petechiæ, and a discoloration and separation of the epidermis, in the advanced stage of the complaint.

The diseases it is likely to be confounded with, are *enlargement of the mesenteric glands*, and hydrocephalus internus. The author also mentions *inflammation of the lungs*, which we should have thought hardly possible, had he not stated that he had known many instances of such a gross blunder.

" It may be distinguished from *enlargement of the mesenteric glands*, by the accession of fever occurring in the latter generally in the evening only; by the patient being more restless at that time, instead of being inclined to sleep, as in remittent fever; by the intestinal evacuations having but little alteration from their natural appearance, that is to say no more generally than what may be supposed to arise from a defective absorption of chyle; by a peculiar mark of distress in the countenance; by the sleep being for the most part undisturbed; and by the length of time the complaint has existed. The fever accompanying enlargement of the absorbent glands in the mesentery, is of a hectic nature, generally terminates with profuse perspiration, and, in every instance that I have seen, has been free from delirium."

If any of our readers think they cannot distinguish the remittent fever from inflammation of the lungs, we refer them to Mr. Coley's treatise, where they will find the distinction very minutely drawn. The disease, we doubt not, is often confounded with hydrocephalus internus: we shall, therefore, quote this part of Mr. Coley's diagnosis.

" The symptoms denoting hydrocephalus internus cannot be confounded with the other disease, until effusion has taken place to such an extent as to compress the brain and impair its functions. In its previous stage a manifest difference must have been observable, from the acuteness of the pain in the head, from the intolerance of light, from the agitation or tossing of the head, and from the absence of

* " 'A Practical Treatise on Various Diseases of the Abdominal Viscera, by C. R. Pemberton, M.D.'—1806. G. and W. Nicol, London."

sleep; sleep, to which must be added, the *healthy* state of the bowels. It might also be suspected that this disease is commencing when the above symptoms have been observed to succeed much irritation about the gums during the formation and evolution of the deciduous teeth; as it is not uncommon for it to arise from the inflammation of the membrane lining the alveolar processes, or of the capsules of the teeth being translated by the operation of sympathy, or some other cause, to the membranes of the brain.

"When effusion has commenced, the symptoms are such as proceed from compression on the brain from other causes, as squinting, interrupted or stertorous breathing,* paralysis generally on one side of the body, insensibility to external stimuli. At length the pupils are dilated and insensible, the pulse intermits, the eye-lids are half closed, the evacuations are involuntary, and in this stage of the disease those from the bowels are often of a greenish or other unhealthy appearance.† The countenance is pale, the muscles of the face are generally distorted, and convulsions often arise and continue from the time the apoplectic symptoms commence, till death closes the scene.

---

* "The patient in this state generally performs about three respirations, and then ceases to breathe for some time, after which respiration commences again for three or four times, and so on. This interruption arises from the comparative insensibility of the lungs, in consequence of a fluid effused on the brain, compressing the origin of the nerves. The circulation of venous blood, which takes place after the oxygen has been all absorbed from the pulmonary air-cells, produces a disagreeable or painful sensation, called suffocation, which rouses the diaphragm and the other inspiratory muscles into action, and a deep inspiration comes on, followed generally by two or three smaller inspirations, by which means the painful sensation is removed. When the pressure on the brain is very great, the heart neglects its duty, which occasions an intermitting pulse. This arises from the sensibility or irritability of that viscus being so much impaired, as to suffer the venous blood to accumulate in the right auricle, till the pressure of it produces a stimulus that excites it into action, and the circulation goes on again."

† "It may seem extraordinary that in the early part of this disease no affection of the bowels should exist, and that it should make its appearance as the original disorder advances. The cause of it is a torpor of the liver and of the stomach and bowels, produced by the pressure of the fluid effused on the brain, and particularly on that portion of the great sympathetic nerve which proceeds from it, and extends to those important viscera. In consequence of this, the healthy secretions of those organs are suspended or obliterated, and the fæces are of nearly the same disordered appearance as in remittent fever. When this occurs exclusively from pressure on the brain, and is unaccompanied with remittent fever, I have always considered it a dangerous, and have generally found it a fatal symptom."

"In this complaint the muttering expressions are incoherent, the screamings are acute and loud, and, as was before observed, the patient cannot be roused to attend to any thing, being like one in a profound sleep. While the sense of pain continues, the hands are constantly carried towards the head.

"In the delirium or stupor of remittent fever, the attention of the patient may be excited for a few moments by strong external impressions, as by talking loudly to him, or by sudden agitations of his body, and there is never any tossing of his head from one side to the other, but, on the contrary, the child is disposed to be still, and to remain in one posture, unless roused by the officiousness and anxiety of his friends or attendants. The face is flushed, and the eye-lids are closed, or, if wide open, they have a foolish disagreeable kind of stare, which is particularly conspicuous in those cases where the patients possess a perfect knowledge of every thing that is going forward, but are unable to articulate. The respiration is quick, but *not interrupted;* and the pulse *never intermits* excepting in cases of extreme debility. There is no squinting; the pupils are sometimes contracted, and sometimes dilated, according to the degree of stupor; and when temporary paralysis happens, it is in those parts which are *subservient to the power of volition.* The hands are seldom carried up towards the head, and when they are, we may perceive that the intention is that of picking the skin about the face, and not that of expressing pain in the head. When convulsions happen, it is impossible during their continuance to distinguish the two diseases, but after they have ceased, if they may have proceeded from remittent fever, the faculties of the patient will be restored.

"The tongue, in both diseases, is furred when the bowels are affected.

"In addition to what has been stated, it may be remarked, that in every case of hydrocephalus internus that has come under my care, I have observed, before any attack of the disease, a peculiar dullness about the eyes, with some dilatation of the pupils, which have appeared to dispose the children to keep the head in a prone position, or to incline it to one side."

For the author's account of the remote causes of the complaint, we must refer to his work. The proximate cause he considers to be a torpor or defective action of some part or of the whole of the chylopöietic system.

"When this has taken place, digestion is at a stand, and the food, instead of being converted into chyle for the nourishment of the body, undergoes a kind of putrefactive fermentation, which is considerably promoted by the heat of the body and accidental constipation. This mass of highly disordered contents occasions considerable irritation in the bowels, which is evinced by the occasional pains, by the itching of the skin on the face, and various other parts, and by the general restlessness of the patient; and if it be not removed, the fever soon commences."

"As remittent fever advances, it has been before remarked that extreme extreme emaciation and debility appear. When we reflect upon the highly disordered state of the bile, if any be secreted, and upon the suspension of the digestive process, we cannot rationally expect that any chyle can be formed, or if that could happen, that it would be in a proper state for the lacteal absorbents to imbibe. As this is the principal source whence the body can derive its nourishment and strength, it must follow of course, when this fails, that emaciation and weakness will present themselves. The only supply, during the long abstinence that obtains in the disease, proceeds from the absorption of fat that may have been accidentally deposited in the cellular substance. After this state has continued many weeks without a return of the healthy functions of the digestive organs, the blood becomes attenuated or in part depraved for want of a supply of chyle, and is effused on various parts of the body; whence in the skin we observe petechiæ, and from the stomach and bowels bloody evacuations."

Whatever opinion may be entertained thus far respecting Mr. Coley's publication, we doubt not that he will receive the thanks of his brethren for communicating a faithful history of his judicious and successful treatment, as well as obtain the more substantial reward of increasing practice and reputation. Having determined the proximate cause of the disease to consist of a disordered action in some part of the digestive organs, the indications of cure, says Mr. Coley,

" Must be to expel from the intestinal canal any irritating materials that may have accumulated, and to excite the disordered parts into a vigorous and healthy action. These purposes may be answered in some measure by the exhibition of purgatives, but more completely and expeditiously by some preparation of mercury, which, when properly administered, is capable of promoting, in a peculiar degree, a healthy state of the viscera, concerned in the formation and absorption of the chyle. As the grand objects to be had in view are the encouraging a secretion of bile, when that is defective, and of the succus gastricus and intestinalis, so we must continue the employment of the mercury until these salutary changes occur. The manner in which I have given this medicine, and the extent to which I have carried it, may, to those who are unacquainted with its utility, appear very unusual. I was first led to the more general use of it by observing the rapid recovery consequent to the administration of a few doses of the oxyde or submuriate, in small quantities, in several cases of this disease, after a long continuance of purgatives had been productive of no benefit. These first cases were such as arose more particularly from a deficient action of the liver only, but I soon found that the same salutary consequence resulted from the employment of this medicine in those depending on disordered action in the bowels. I was the more pleased on observing the beneficial effects of the practice, on account of the frequent failures I met with by pursuing the usual mode of treating the disease only with aperient medicines. By that plan I lost many patients; but since I have adopted the other mode of practice, although I have had hundreds of cases under my care, the complaint has not terminated fatally, excepting in one instance, where effusion took place in the brain, constituting hydrocephalus internus, in consequence of extreme debility from a previous disease.

"The intention with which most practitioners exhibit mercury in this disorder, is that of exciting a strong peristaltic action in the intestines;* but it will be found to exercise a still more speedy and beneficial influence when given with the view of its being absorbed into the system, when it rouses the liver to secrete its bile, the stomach to prepare the gastric juice for the purpose of digesting the food, and the intestines to convert that digested food into chyle, for the nourishment and repair of the enfeebled and emaciated frame. With this design I have in general continued it nearly through the whole of the illness, and have frequently, when it was requisite to produce a purgative operation, given four or five grains of the submuriate every other day, or oftener, for several weeks in succession, not only without any inconvenience, but with the most manifest advantage. Although my practice has been so bold and decisive, I have not met with a single instance of ptyalism nor of mercurial erythema; and the debility consequent on the evacuations has been incomparably less than would have resulted from the natural progress of the disease, or the common mode of treating it. That a return of the healthy action of the digestive organs, a circumstance most desirable in this complaint, may with justice be attributed to the agency of this medicine, I think this fact will testify, namely, that in some very obstinate cases, in which, either from defective absorption, or from its passing off too rapidly from the bowels, it has not produced any salutary change after a long period of time, I have been induced to give half a grain or a grain of the submuriate, or a smaller dose of the oxyde, twice daily, which, at the end of three or four days, has generally effected a secretion of bile, removed the unnatural appearance of the fæces, and been speedily followed by a re-establishment of health.

"In simple cases of this disease, I know that the employment of purgatives alone will succeed; but in most of the cases requiring my assistance, there has been such an extreme torpidity of the chylopöietic viscera, as to render the use of mercurials necessary. In these instances, nothing with which our art can furnish us, could have saved the patients from destruction, had not the most vigorous measures and firm conduct been pursued.

"As soon as I have visited a patient ill with this fever, I direct a dose of hydrargyri submurias to be given, containing from one to five grains, according to the age and constitution of the patient, the severity of the attack, or the state of the bowels. Two or three hours having elapsed, a draught composed of sulphate of magnesia, tincture of jalap, and infusion of senna, is administered, and repeated every two hours, until a copious evacuation takes place from the bowels,

* "Hamilton. Pemberton. Butter."

which which I always carefully inspect. After this, the same dose of the submuriate is repeated every second night, and the draught the following morning, so as to produce several evacuations, until it be ascertained that the digestive organs have regained their natural energy. This effect will sometimes happen in a few days; at others several weeks will elapse before any favorable change will occur. If the constipation be very great, the aperient draught should be repeated every morning, or a dose or two of sulphate of magnesia, or of any other neutral aperient salt, so as to occasion one or two evacuations daily from the bowels. When the fæces have become healthy, they are found to be of moderate consistence, having some impressions of the larger intestines upon them; to be of a yellow color, resembling powder of rhubarb; and free from mucus and all other matter that is unnatural to them. As long as they seem to have undergone no change in their passage through the cæcum, colon, and rectum, which is known by their fluidity and heterogeneous consistence, I direct the mercury to be given in a *large* dose in conjunction with, or followed by, a purgative, in the manner above described; but after the bowels have resumed their functions, and the only alteration in their contents is found to consist of a preternatural appearance as to color, I order the former medicine to be repeated once in twelve hours, or oftener, according to the urgency of the case, in small doses; by which means, at the end of a few days or a week a secretion of bile takes place, and the disease is entirely removed."

Having already far exceeded the space we had intended this small volume should occupy, we must close our notice of it without inserting either more particulars of the author's practice, or citing any of the cases which he has selected for publication. The book is worthy of perusal.

---

### *Edinburgh Medical and Surgical Journal, No.* XXXIV.

### I. *Singular Case of Lithotomy, performed on a Man who had attempted to saw and break down the Stone in the Bladder. By* John Rodman, M.D.

This very distressing case of calculus occurred to a man of singular idiosyncracy, who became and continued very corpulent on small quantities of meagre vegetable diet. Soon after the operation of lithotomy was with great difficulty performed on him, calculi again began to form; and in about a year from the operation, it became necessary to cut into the urethra in the perinæum to extract a calculus lodged there. In three months after this, it became evident that a stone was impacted about the neck of the bladder; and the novel part of the detail rests on the employment of a file and a boring instrument to diminish the size of this stone. These instruments were applied to the calculus, through through the opening in the perinæum. For a time the patient appeared to be relieved by this contrivance ; but died some weeks after, of mortification of the bladder and intestines.

II. *Observations of the different Hypotheses published to account for the Effects of the Wind of a Ball.*

This anonymous writer considers the opinion of the mischievous effects arising from the " Wind of a Ball," to be the offspring of ignorance and superstition ; and he acutely observes,

" Did the wind of a shot, the tremor of the atmosphere, the accumulation of electricity, or the developement of any subtile matter by the flight of a ball, produce such fatal consequences, it is easy to perceive they must be equally numerous with the wounds which are received by actual contact. For, although it is not susceptible of arithmetical calculation, yet it may be justly inferred, that when two vessels fire right into each other, as many balls pass within a hair-breadth as those that strike men on-board. How, then, does it happen that, among the hundreds and thousands that strew the decks after a long and severe engagement, only one, two, or three, are found without marks of injury? If the principle operates in one instance, it must in all. All the effects arising from mechanical violence are seen and experienced, but those originating from this unknown, or these unknown causes, are heard of only once in ten thousand times."

It does, indeed, appear incomprehensible, if there be such a property or principle as the wind of a ball, possessing the efficient power of destroying life so suddenly, that it should so seldom act, seeing that, in every engagement, so many must come within its sphere of influence. The deaths occurring in battle where no external violence is detectable, are referred to extreme emotion and agitation of mind, by this writer; and the opinion is supported by analogical facts recurring in civil life from paroxysms of passion.

III. *Observations on the Fever prevalent in the Mediterranean, as it occurred on-board one of his Majesty's line-of-battle Ships on that Station.*

The writer of this paper had been stricken, he says, *à priori*, with the notion that there was something in the climate of the Mediterranean station peculiarly predisposing to inflammation: and his practice and success in the " nearly one hundred cases" of which he gives the result, we cannot doubt confirmed his preconceived opinion.

In the fever here described, the cold stage was short ; prostration of strength, violent head-ache, severe pain at the scrobiculus cordis, and very commonly in the thorax, sometimes times with dypsnœa, but seldom cough, quickly succeeded the rigor. Full and hard pulse, increased heat, flushed face, rough and dry tongue, and constipated bowels, made up the catalogue of symptoms. Great thirst and irritability of the stomach seldom were present.

This fever occurred in the hot autumnal months, while the ship was at anchor in Port Mahon.

" Blood-letting was the remedy principally relied on, and the extent to which it was carried was regulated by the patient's feelings, viz. either until a remission of pain or incipient deliquium took place. Before this was effected, 50 or 60 ounces were frequently abstracted by a large orifice, and, if the pain recurred, I did not hesitate to repeat the evacuation to the extent of 20 or 30 ounces more, within four-and-twenty hours from the first attack; at least this was the practice I latterly followed, and I found it successful. When thus freely employed on the first day, there was seldom occasion to have recourse to venesection beyond a third time."

In the " nearly one hundred cases" three only were fatal, in one of which the symptoms of gastritis were strongly marked; in the other two there was great determination to the brain.

" The three fatal cases alluded to above, were the three first that came under my care, and although, in compliance with the advice of the then physician to the fleet, I had recourse to blood-letting, yet, not being thoroughly aware of the nature of the epidemic, I have reason to regret that I did not, early enough, carry that powerful remedy to the extent that I afterwards found I might do with advantage and safety. Indeed, if six-and-thirty hours are allowed to elapse without due evacuations, the most favorable time is gone, the inflammatory action much increased, and determination to some of the viscera already begun;—blood-letting becomes more than ever necessary, but the patient is less able to bear it.

" In addition to venesection, a moderate purging was kept up by calomel, conjoined with antimony, and assisted by small doses of the saline purgatives, largely diluted, having found that in this way they sat well on the stomach.

" In three cases, where there was no evident affection of the thorax or abdomen, and where the heat was unusually great, the cold affusion was employed. In all the three it procured great temporary relief; but in one, the disease terminated by an extensive abscess, situated over the gluteus maximus, and the other two were attacked, on the succeeding day, by severe pains in the thorax, which were relieved by the liberal use of the lancet.

" There occurred also six or seven cases, apparently slight, and in which, consequently, evacuations were more sparingly employed, but they eventually proved the most troublesome, as the fever terminated in, or was succeeded by, disease of the liver or spleen. Four of these were sent to the hospital, and I do not know their fate; the others recovered on-board, after a long course of mercurial preparations."

In properly discriminated cases of fever, with great increase of vascular action, and inflammatory determination to particular organs, what doubt can remain of the propriety of blood-letting, proportioned in degree to the peculiarities of individual instances. But by too much generalization and too little attention to the phenomena of individual cases, the phlebotomists and anti-phlebotomists have each fallen into important and alarming errors. The rational practitioner is guided in the management of any case by the peculiarities of that case, independent of hypothesis. A nice knowledge of the quality of the deviations from the actions of health, can alone direct in the choice and application of remedies. A predilection for venesection induces one party to call for more blood on all occasions; while the other, equally wedded to the notion of preventing debility, generally rejects or employs it in too trifling a manner to be useful. If the former mistake because they do not reason, the latter fall into error against reason. The most thoughtless empiricism has not gone further than the first, and the second, by supporting the increased morbid action, actually plunge their patients into the hazardous debility they labor to avoid. "The rapidity with which the patient recovered his strength, on the removal of the febrile action," says the writer of this paper, "notwithstanding those large evacuations, was, for a long time, matter of astonishment to me." In all instances of indirect debility, moderating the increased morbid action, reduces in the same ratio the chance of subsequent weakness. This axiom the reasoners have lost sight of, when they fear to use the means of reducing increased action on the apprehension of future debility.

(To be continued.)

